# High-Intensity Focused Ultrasound Ablation of Uterine Fibroids: A Review

**DOI:** 10.7759/cureus.44680

**Published:** 2023-09-04

**Authors:** Nainita Patel, Kamlesh Chaudhari, Dharmesh Patel, Jalormy Joshi

**Affiliations:** 1 Obstetrics and Gynaecology, Jawaharlal Nehru Medical College, Datta Meghe Institute of Higher Education and Research, Wardha, IND

**Keywords:** fertility sparing treatment, ultrasound, thermal ablation, premenopausal age, fibroid

## Abstract

Leiomyomas, or uterine fibroids, are growths consisting of muscle and tissue that develop in or on the uterine wall. The most frequent benign uterine tumours in women of reproductive age are thought to be fibroids. Dysmenorrhea, spotting, hypermenorrhoea, abdominal pain, pressure on surrounding organs, and issues with micturition and defecation are among the symptoms that are often present. Fibroids can form as a single nodule or as a cluster. Uterine fibroids, especially large submucosal and intramural uterine fibroids, can cause obstacles to implantation and lead to pregnancy loss. Uterine fibroids can be treated without surgery and with little downtime using focused ultrasound. There is published research showing that women can conceive and have healthy children after therapy, thus protecting fertility. The ablation of uterine fibroids by high-intensity focused ultrasound (HIFU) is successful since the volume of the fibroids is significantly reduced.

## Introduction and background

Benign uterine tumours known as fibroids typically occur in premenopausal women. Uterine fibroids are benign, hormone-sensitive, smooth muscle tumours; depending on age, the incidence in women of childbearing age has been reported to be as high as 40% [1]. A recently created non-invasive technique called high-intensity focused ultrasound (HIFU) employs ultrasound probes to concentrate HIFU pulses on certain fibroids [2]. Given that uterine fibroids can harm fertility, their existence significantly affects women who may want to get pregnant. Uterine fibroids have been linked to both sterility and an increased risk of pregnancy problems [3-5].

The size and location of the fibroid will determine whether a hysteroscopy or open abdominal surgery is the best course of action. Conservative medical approaches, such as the administration of gonadotrophin-releasing hormone (GnRH)-analogues, progesterone-containing oral contraceptives, etc., can alleviate symptoms related to the fibroid, but they can delay the pregnancy and frequently have only short-term effects [6].

As a result, non-invasive gynaecological procedures are receiving more attention as a way to reduce surgical morbidity and maintain fertility. Robotic-assisted laparoscopic myomectomy (RALM) is one of the most advanced minimally invasive options, offering impressive three-dimensional and magnified visualization capabilities, natural, finger-like, and intuitive control of surgical instruments, and superior ergonomics. In patients with smaller myomas, RALM is proven to be superior to conventional laparoscopic myomectomy (CLM) and is associated with reduced intra-operative bleeding while CLM is superior in cases with higher-weight myomas, as shown in a meta-analysis by Tsakos et al. However, for the rest of the parameters like loss of blood, duration of operation, and complications, similar performances were recorded by RALM and CLM according to the above study, while another method also being performed for fibroids, abdominal myomectomy was shown to be inferior to RALM in all parameters except for operation duration [7].

One another non-invasive method is HIFU. With the use of this technology, ultrasonic wave beams are concentrated at a small target volume and delivered to tissues located deep inside the body. Without causing harm to nearby and surrounding essential structures, this extracorporeal source of concentrated ultrasound radiation can produce thermal coagulative necrosis [8-11]. Under the direction of magnetic resonance imaging (MRI) or ultrasound, HIFU is used as a non-invasive treatment method to selectively ablate fibroids, even those with a diameter of less than 2 cm, without causing harm to nearby structures [12,13]. Because of its outstanding therapeutic efficacy and low to no pain level, it can be done as an outpatient surgery [14-17].

HIFU has been a more popular alternative in the last 20 years for treating uterine fibroids and adenomyosis. It has been proven to be both secure and efficient. The fertility and pregnancy rates after HIFU show encouraging results in systematic reviews and meta-analyses, making it a desirable alternative for women seeking fertility [18].

The intent of this review is to assess the effectiveness of HIFU in treating patients with uterine fibroids so that healthcare professionals can use the procedure and help improve the quality of life and reproductive health of women.

## Review

Methodology

**Figure 1:**
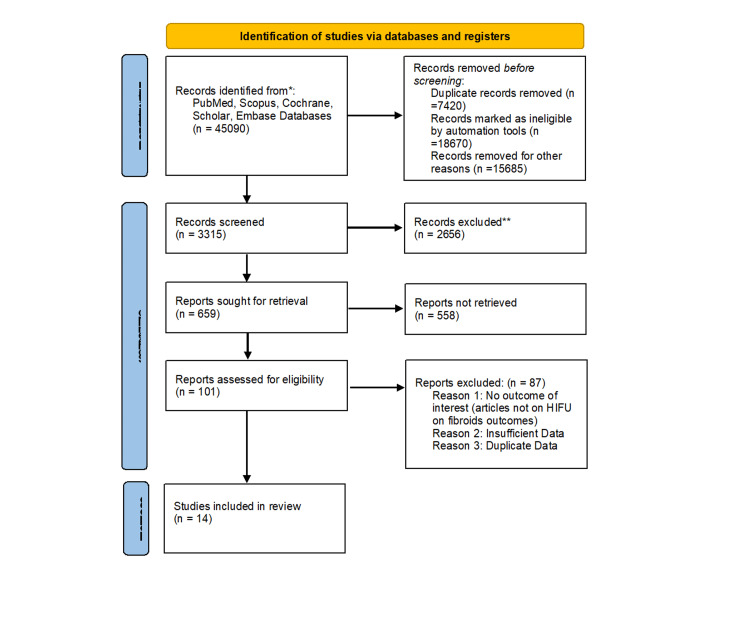
The PRISMA (Preferred Reporting Items for Systematic Reviews and Meta-Analyses) flow diagram illustrates the process of study selection

From the commencement of the database up through July 2023, the authors found qualifying papers in the PubMed, Medline, Embase, and Cochrane Library databases. In the abstract or full text of the literature review, the following keywords were used: (HIFU OR "high-intensity focused ultrasound" OR "high intensity focused ultrasound") AND (leiomyoma* OR fibroid* OR myoma*). Additionally, a manual search of the literature was done using the references of studies that were already published. Studies that were published in English, studies that included uterine fibroid-affected women, clinical trials, comparative studies, observational studies, and studies that included quantitative data of outcomes of interest i.e. effectiveness of HIFU in uterine fibroid patients, and its outcome in pregnancy, and studies that compared HIFU with other surgical treatments for uterine fibroid-affected women were all considered for inclusion. Studies that aimed to compare HIFU with percutaneous microwave ablation or pharmacological treatment, research for which the original datasets were not available, and studies published as letters, comments, case reports, or literature reviews were all excluded. Figure 1 shows the PRISMA flowchart [19].

Results

Table 1 shows the characteristics of the included studies. About 14 studies were included in this review, out of which five studies were retrospective and five were comparative studies as well as one multicenter cohort study, one clinical trial, one prospective study, and one observational study. All different studies had different outcomes but all were related to uterine fibroids as well as the efficacy and subsequent pregnancy rates post-HIFU. 

**Table 1:** Characteristics of Included Studies QoL: quality of life; L: large; S: small; USS: uterus sparing surgery; LM: laparoscopic myomectomy; FV: fibroid volume; USg-HIFU: ultrasound guided high intensity-focused ultrasound

Author Name	Study design	Technique used	Outcome of interest	Result
Jindal et al [20]	Retrospective study, 167 patients	USgHIFU	Fibroid volume (FV), SSS, QoL	Reduction in FV 68% and 75% at 6,12 months, improved QoL, SSS; P < 0.001
Lyon et al. [21]	Observational study, 12 patients	USgHIFU	FV, QoL, Complications, SSS	Reduction in FV 51.9 ± 11.1 %, standard deviation (SD), SSS 40.6 ± 32.7 SD, at 24 months (p < 0.005)
Chen et al. [22]	Multicentre cohort study, 2411 patients 1353 (HIFU)	USgHIFU	Complications, return to normal activities, hospital stay, QoL	Complications 02%, QoL improved; P = 0.001 at 6 months, hospital stay median time 8 days (interquartile range, 7–10 days).
Liu et al. [23]	Comparative study 166 patients	USgHIFU and LM	Efficacy, complication, and QoL between two techniques	Efficacy (P > 0.05), QoL same for two groups, lesser complication in HIFU patients
Liu et al. [24]	Comparative study, 188 women	USgHIFU and secondary myomectomy	Symptom alleviation, re-intervention, adverse effects	Fewer adverse events in the HIFU ablation group P = 0.01, cumulative risk for re-intervention after HIFU ablation is lower compared to myomectomy
He et al. [25]	Retrospective study, 81 women	USgHIFU	Shrinkage rate, symptom relief, QoL	Average volume reduction rate of fibroids 52.5 ± 36.3%, six months after HIFU, Decreased UFS score, and increased QoL
Jiang et al. [26]	Retrospective study, 346 patients	USgHIFU and LM	Compare Pregnancy Outcomes in uterine fibroids patients	Shorter pregnancy interval for HIFU compared to LM 10 months VS. 13 months, p
Wu et al. [27]	Comparative study, 676 patients	USgHIFU and LM	Pregnancy Outcomes in uterine fibroid patients	68.4% of women became pregnant after USgHIFU and 66.7% after LM, rate of cesarean delivery was lower in the USgHIFU group at 41.6% (p < 0.05)
Jeng et al. [28]	Retrospective study, 500 patients	USg-HIFU	FV, QoL, pregnancy Outcomes, Adverse effects	Lesion size reduced by 40.2% after 3 months of HIFU, QoL improved, and pregnancy was reported in 12 patients
Vincent et al. et.al [29]	Prospective study, 20 patients	USg-HIFU	FV, QoL, Adverse effects	FV reduction was 46.9 (range -8.8-73.1) at 1 month, UFS-QOL scores were reduced by 40.7% at 3 months; no complications encountered
Wang et al. [30]	Retrospective comparative study, 245 patients	USg-HIFU and Uterus Sparing Surgery (USS)	Clinical outcomes like recurrence rate, QoL, complications	symptom relief rate was 95.9% for HIFU, decreased recurrence rate for HIFU compared to USS, no major complications noted in HIFU
Lee et al. [31]	Clinical trial, 36 patients	USg-HIFU	FV, symptom improvement, QoL, safety	Mean FV reduction was 45.1% at 5 months after HIFU treatment, Symptoms and QoL improved after HIFU, with no complications related to symptoms and safety.
Ren et al. [32]	Comparative study, 587 patients	USg-HIFU and LM	Safety and clinical efficacy	Intra-operative blood loss is reduced in the HIFU group, with lower complications than LM group; FV decreased significantly at 12 months p < 0.05
Chang et al. [33]	Retrospective study, 107 patients	USg-HIFU	FV, QoL, symptoms in patients of large (L) and small (S) Fibroid	Significant reduction in FV in L and S group but higher in S group p < 0.05, improved QoL in both groups

Discussion

In order to determine the efficacy and safety of HIFU in the treatment of uterine fibroids and adenomyosis, 167 patients who underwent the procedure between July 2018 and December 2020 were included in a retrospective analysis. All patients with single or multiple fibroids received HIFU treatment in this retrospective analysis. A gynaecologist examined each patient, obtained pertinent gynaecological and medical history, and used the uterine fibroid symptom and QOL questionnaire (UFSQOL) to evaluate symptoms. They discovered improvements in symptoms such as menorrhagia, discomfort, pressure symptoms, urine symptoms, and quality of life scores, as well as a reduction in fibroid volume of around 68% and 75% at six and 12 months respectively. Reintervention rates after HIFU were 7.7%, and six study participants reported successful pregnancies after the procedure [20].

A single-centre prospective observational study was conducted in the UK. Symptomatic uterine fibroid patients who were referred to the HIFU unit but declined routine surgical or radiological intervention were considered for treatment with USGHIFU. Before and periodically after treatment, clinical evaluation and monitoring of adverse events and fibroid symptoms as well as health-related QoL questionnaires (UFS-QOL), and MRIs were carried out to evaluate patient outcomes. It has been noted that 12 of the 22 patients underwent HIFU ablation, eliminating a total of 14 fibroids, and received a two-year follow-up. No major side effects were noted, but one patient experienced second-degree skin burns. Three months, 12 months, and two years after treatment, the mean symptom severity scores (SSS-QOL) significantly improved. Their research indicates the low risk of complications and clinical effectiveness of HIFU for uterine fibroids [21].

In order to determine the clinical results of HIFU and surgery in the treatment of fibroids, a prospective cohort study was conducted. In total, 472 hysterectomies, 586 myomectomies, and 1353 HIFUs were performed on women. After HIFU, both uterine fibroid symptoms and QoL improved more quickly than outcomes with surgery. They found that patients who underwent HIFU had a comparable longer-term QoL to surgery and significantly lower morbidity [22].

In order to examine the clinical efficacies of HIFU and laparoscopic myomectomy (LM) for treating fibroids and their impact on patient's QoL, a non-randomized control trial with 166 patients with uterine fibroids was done. The findings of a one-year follow-up revealed that the patients of the HIFU group had a total efficacy rate of 97%, whereas 67 patients with uterine fibroids underwent operations. The efficacy rate between the two groups did not significantly differ. Both therapies successfully raised patients' quality of life. HIFU group patients, in contrast to the LM group, had a negligible loss of blood, reduced hospital stay, and minimal side effects; this variation was statistically significant [23].

Around 188 women with recurring uterine fibroids (symptomatic) after myomectomy were included in the retrospective analysis to examine the prolonged symptom relief and re-intervention of HIFU ablation and secondary myomectomy. They discovered that the follow-up time was equivalent for the two groups of women who received secondary myomectomy and HIFU ablation and that the HIFU ablation group experienced fewer adverse events than the myomectomy group. They found that HIFU ablation of recurring symptomatic uterine fibroids provided comparable prolonged symptom relief with a gap of a relatively long time before re-intervention and fewer side effects than other treatment options [24].

An evaluation of the shrinkage rate, symptom relief, and QoL improvement following ultrasound-guided HIFU for multiple uterine fibroids, involving 81 women with multiple fibroids, was conducted retrospectively. MRIs and the UFS-QOL questionnaires (for uterine fibroid symptom and QoL) was used to assess the patient. One, three, and six months following HIFU treatment, they discovered that the UFS score had greatly lowered, the QOL score had significantly improved, and the fibroid volumes had significantly decreased. They found that treating patients with numerous uterine fibroids with HIFU is secure and efficient [25].

An analysis of the pregnancy outcomes of individuals with uterine fibroids following HIFU ablation and LM was done in a retrospective study. In total, 346 patients with uterine fibroids who wanted to get pregnant made up the trial group; 152 of them underwent HIFU ablation therapy, and 194 underwent LM therapy. In a follow-up period of 42 months following therapy, the pregnancy outcomes were assessed, the baseline characteristics of the patients were noted, and the differences between the two groups were contrasted. They discovered that the uterine fibroid patients in the HIFU category had a noticeably shorter pregnancy interval than those in the LM group. In comparison to surgical treatment, the benefits of HIFU treatment for uterine fibroids included fewer problems, fewer numbers of days spent in the hospital, reduced postoperative pain, quick recovery, and considerably enhanced the QoL of patients. They discovered that the interval of pregnancy was much shorter for patients who had undergone HIFU compared to LM patients. The benefits of HIFU treatment for patients with fibroid include fewer problems, shorter hospital stays, reduced pain post-procedure, quick recovery than surgical treatment, and considerably enhanced QoL of patients [26].

To assess the results of pregnancies following USGHIFU ablation against LM, a comparative study involving 676 women with symptomatic uterine fibroids was carried out. In total, 336 patients had LM treatment, while 20 patients received HIFU treatment. After ablation, 219 women were pregnant, and 224 women became pregnant after LM. The HIFU group had a higher rate of spontaneous vaginal births and a decreased rate of caesarean sections as compared to the LM group. In this way, HIFU ablation also helps lower the rate of caesarean deliveries. After HIFU, there are significantly fewer cases of placenta previa, placenta increta, caesarean birth, and postpartum haemorrhage than after LM [27].

A cross-sectional analysis was performed retrospectively on 404 patients with uterine fibroids. Utilizing self-reported questionnaires, secondary outcomes such as QoL, pregnancy rate, and harmful adverse effects were assessed following HIFU treatment of the patients' uterine fibroids. They discovered that the lesion size decreased by 40.2% three months after receiving HIFU treatment for uterine fibroids. At three months after treatment, patients with uterine fibroids who received HIFU reported noticeably better QoL ratings, pain scores, sexual satisfaction, and symptoms associated with compression. It is a realistic therapy option with the benefits of symptom relief, non-invasiveness, fewer side effects, quick recovery, and pregnancy preparation [28].

To determine the therapeutic effectiveness and safety of ultrasound-guided HIFU in treating uterine fibroids, prospective cohort research was conducted. Around 20 women with symptomatic fibroids received HIFU with ultrasound guidance. Following therapy, volume reduction of fibroid was 46.9% at one month, 57.4% at three months, 60.1% at six months, and 75.9% at 12 months. At three months, six months, and 12 months after therapy, the modified UFS-QOL ratings were lower by 40.7%, 45.5%, and 44.9%, respectively. Nothing particularly difficult came up. Using ultrasound-guided HIFU to treat symptomatic uterine fibroids appears to be both efficient and safe [29].

In order to evaluate the prolonged clinical outcomes of uterus-sparing surgery and US-guided HIFU ablation for the treatment of fibroids located submucosally with symptoms, a retrospective study including 245 women was carried out. The rates of symptom alleviation, recurrence, and significant complication incidence were compared between the two groups. The rate of symptom remission for uterus-sparing surgery was 89.1%, compared to 95.9% for HIFU ablation. For US-guided HIFU ablation, the cumulative symptom recurrence rate was 1.7%, 6.8%, 9.4%, and 11.9% at one, three, five, and eight years, respectively. HIFU ablation exhibited a statistically reduced rate of symptom recurrence and a better rate of symptom alleviation when compared to the uterus-sparing surgery group. Around 3.1% of patients who underwent uterus-sparing surgery experienced serious complications. In the group receiving HIFU ablation, no significant problems occurred. Their research demonstrated that for treating symptomatic submucosal fibroids, the prolonged outcomes of HIFU ablation might be superior to those of surgeries that are uterus-sparing. HIFU ablation with US guidance may also be less dangerous than uterus-sparing surgery [30].

A prospective clinical trial examined the effectiveness and safety of a novel portable USGHIFU with cutting-edge targeting and beam steering technology for the treatment of uterine fibroids. From November 2013 to November 2015, 36 subjects with a total of 59 uterine fibroids were enrolled. Every participant received HIFU therapy together with 3D electronic guidance. Prior to HIFU, right after HIFU, and one, three months, or five months following the treatment, MR imaging tests were conducted. Analysis was done on the fibroid volume shrinkage (FVS), non-perfused volume ratio (NPVR), symptom relief, quantified life quality assessment, and safety. The size of the treated uterine fibroids ranged from 7.5 to 274.4 cm3. Following HIFU therapy, there were significant improvements statistically in quality of life and symptoms associated with uterine fibroids. There were no notable safety-related or problematic symptoms. In the lengthy follow-up, 78.8% of those polled expressed satisfaction with their HIFU therapy. According to the above clinical trial, a portable USGHIFU with expanded functionalities may safely and effectively treat uterine fibroids [31].

A contrastive study examined the safety and therapeutic effectiveness of LM and HIFU, two therapies for uterine fibroids. From 587 patients with uterine fibroid, clinical information was gathered. The patients were divided into two groups: 287 cases received HIFU treatment, and 300 received LM treatment. Progression-free survival (PFS) was taken as the main outcome. Secondary outcomes were operation results, (time of procedure, blood loss, fluid replacement), haemoglobin level post-surgery, and clinical competence. Additionally, in patients who underwent HIFU, fibroid volume before therapy and three, six, and 12 months following treatment were also examined. They discovered that the observation group's (patients who received HIFU) operating time was cut and that the intraoperative blood loss and fluid replacement were remarkably decreased. The total effective rate of the patients who received HIFU was 98.6% and 95.3% for those who underwent LM, and the difference was statistically significant. Regarding problems, it was clear that the HIFU group experienced less bleeding and infection than the LM group. Comparisons of the volume of fibroid before treatment and at every three months of interval till one year after surgery in the observation group revealed that the volume of fibroid dramatically decreased. In terms of complications, the bleeding and infection were obviously lower in patients of the HIFU group than it was in the LM group. Comparisons of the fibroid volume before treatment and after every three months following surgery in the observation group revealed a significant reduction in fibroid volume [32].

A prospective study comparing HIFU treatment for type I and type II submucosal fibroids was conducted on 55 individuals. Type I submucosal fibroids were found in 27 individuals, while type II submucosal fibroids were found in 28 patients. After receiving HIFU therapy, each patient underwent follow-up exams at one, six, and 12 months. Unfavourable outcomes were also noted. They discovered that patients with type II submucosal fibroids had considerably larger NPV ratios of fibroids than individuals with type I submucosal fibroids. Following HIFU treatment, the fibroid-related symptoms were alleviated. The most prevalent symptom in their patients, menorrhagia, was observed in 74.1% of group type I patients and 67.9% of type II fibroid patients. In one HIFU session, with an average operating room time of less than 90 minutes, both types of submucosal fibroid could be successfully removed [33]. Similarly, a retrospective study utilized information from patients who underwent ultrasound-guided HIFU treatment for fibroids between April 2015 and April 2019. Here, 107 patients having fibroids were split into two categories, i.e. one being the S-type category characterized by smaller fibroids (less than 10 cm in size), and the other being the L-type category characterized by bigger fibroids (more than 10 cm in size). They employed MRI to compare the uterine and fibroid volumes prior to and after three months of HIFU ablation to assess its effectiveness. A visual analogue scale and a fibroid symptom health-related QoL questionnaire were utilized in the three-month follow-up clinical visit to assess clinical symptoms. Uterine and fibroid volumes were dramatically reduced in both the L and S groups, but the pace was noticeably higher in the S group. Clinical symptoms also improved in both groups, but there was no notable difference was seen statistically. Large and small fibroids were reduced in size by HIFU, however, small fibroids with a diameter of less than 10 cm benefited the most. The QoL and symptoms of dysmenorrhea improved in both the L and S groups [33,34].

## Conclusions

Surgery is inferior to HIFU ablation in terms of symptomatic alleviation, QoL improvement, recovery, and serious consequences. In terms of symptom recurrence, re-intervention, and pregnancy rates, HIFU has outcomes that are equivalent to surgery, showing that it is a promising non-invasive therapy that does not appear to worsen fertility compared to surgical procedures for women with fibroids. HIFU was also linked to decreased morbidity and a shorter duration of stay than traditional treatment techniques, including surgery. The capacity of HIFU to preserve fertility is one of its alleged advantages. After a myomectomy, patients run the risk of intrauterine adhesions, uterine rupture, miscarriage, and premature birth, all of which can negatively impact pregnancy prospects or outcomes. Patients are sometimes advised to postpone conceiving for 6 to 12 months to allow the myometrium time to recover. This period may be important for people who want to have children, especially in the case of older expectant mothers. HIFU maximises the window for conception in this group since it has a shorter downtime.
